# Climate Change and Risk of Leishmaniasis in North America: Predictions from Ecological Niche Models of Vector and Reservoir Species

**DOI:** 10.1371/journal.pntd.0000585

**Published:** 2010-01-19

**Authors:** Camila González, Ophelia Wang, Stavana E. Strutz, Constantino González-Salazar, Víctor Sánchez-Cordero, Sahotra Sarkar

**Affiliations:** 1 Laboratorio de Sistemas de Información Geográfica, Departamento de Zoología, Instituto de Biología, Universidad Nacional Autónoma de México, Coyoacán, México; 2 Department of Geography and the Environment, University of Texas at Austin, Austin, Texas, United States of America; 3 Biodiversity and Biocultural Conservation Laboratory, Section of Integrative Biology, University of Texas at Austin, Austin, Texas, United States of America; 4 Laboratorio de Análisis Especiales, Departamento de Zoología, Instituto de Biología, Universidad Nacional Autónoma de México, Coyoacán, México; Yale University, United States of America

## Abstract

**Background:**

Climate change is increasingly being implicated in species' range shifts throughout the world, including those of important vector and reservoir species for infectious diseases. In North America (México, United States, and Canada), leishmaniasis is a vector-borne disease that is autochthonous in México and Texas and has begun to expand its range northward. Further expansion to the north may be facilitated by climate change as more habitat becomes suitable for vector and reservoir species for leishmaniasis.

**Methods and Findings:**

The analysis began with the construction of ecological niche models using a maximum entropy algorithm for the distribution of two sand fly vector species (*Lutzomyia anthophora* and *L. diabolica*), three confirmed rodent reservoir species (*Neotoma albigula*, *N. floridana*, and *N. micropus*), and one potential rodent reservoir species (*N. mexicana*) for leishmaniasis in northern México and the United States. As input, these models used species' occurrence records with topographic and climatic parameters as explanatory variables. Models were tested for their ability to predict correctly both a specified fraction of occurrence points set aside for this purpose and occurrence points from an independently derived data set. These models were refined to obtain predicted species' geographical distributions under increasingly strict assumptions about the ability of a species to disperse to suitable habitat and to persist in it, as modulated by its ecological suitability. Models successful at predictions were fitted to the extreme A2 and relatively conservative B2 projected climate scenarios for 2020, 2050, and 2080 using publicly available interpolated climate data from the Third Intergovernmental Panel on Climate Change Assessment Report. Further analyses included estimation of the projected human population that could potentially be exposed to leishmaniasis in 2020, 2050, and 2080 under the A2 and B2 scenarios. All confirmed vector and reservoir species will see an expansion of their potential range towards the north. Thus, leishmaniasis has the potential to expand northwards from México and the southern United States. In the eastern United States its spread is predicted to be limited by the range of *L. diabolica*; further west, *L. anthophora* may play the same role. In the east it may even reach the southern boundary of Canada. The risk of spread is greater for the A2 scenario than for the B2 scenario. Even in the latter case, with restrictive (contiguous) models for dispersal of vector and reservoir species, and limiting vector and reservoir species occupancy to only the top 10% of their potential suitable habitat, the expected number of human individuals exposed to leishmaniasis by 2080 will at least double its present value.

**Conclusions:**

These models predict that climate change will exacerbate the ecological risk of human exposure to leishmaniasis in areas outside its present range in the United States and, possibly, in parts of southern Canada. This prediction suggests the adoption of measures such as surveillance for leishmaniasis north of Texas as disease cases spread northwards. Potential vector and reservoir control strategies—besides direct intervention in disease cases—should also be further investigated.

## Author Summary

We explored the consequences of climate change for the spread of leishmaniasis in North America. We modeled the distribution of two sand fly vector and four rodent reservoir species found in northern México and the southern United States. Models were based on occurrence data and environmental and topographic layers. Successful models were projected to 2020, 2050, and 2080 using an extreme (A2) and a conservative (B2) future climate scenario. We predicted potential range shifts of vector and reservoir species varying assumptions about dispersal ability and capacity to persist in habitats with different degrees of ecological suitability. Even with the most conservative assumptions the distributions of both vector and reservoir species expand northwards, potentially reaching as far as southern Canada in the east. Assuming that at least one vector and one reservoir species must be present for a parasite cycle, the extent of this shift is predicted to be controlled by the availability of suitable habitat for sand fly vector species. Finally, we computed the human population potentially exposed to leishmaniasis because of these range shifts. Even in the most optimistic scenario we found that twice as many individuals could be exposed to leishmaniasis in North America in 2080 compared to today.

## Introduction

Leishmaniasis is a vector-borne parasitic disease endemic in most tropical regions of the world with approximately two million new human cases reported each year [1],[2]. In the Americas, parasite species belonging to the genus Leishmania are responsible for different clinical pathologies, including the deadly visceral form caused by *Leishmania chagasi*, as well as the mucocutaneous (MCL), localized cutaneous (LCL), pseudo-diffuse (PDCL), and diffuse (DCL) disfiguring forms of the disease caused by at least fourteen Leishmania species from the subgenera Leishmania and Viannia [3]–[6]. Mucocutaneous leishmaniasis is caused by *L. brasiliensis*, *L. panamensis* and *L. guyanensis* [7],[8], while diffuse forms have been related to *L. m. mexicana*, *L. amazonensis* [8], *L. pifanoi*, *L. guyanensis* [6],[9],[10], and *L. panamensis* [7],[11]. Which clinical form is manifested depends on both host immune capacity and the parasite species or strain responsible for the infection even though the genetic determinants of the variation between them remain unknown [6],[12].

In North America (México, United States, and Canada), the transmission of the disease depends on female blood-feeding sand fly vectors from species belonging to the genus Lutzomyia (Diptera: Psychodidae: Phlebotomidae) with several mammal reservoir species serving as parasite hosts; humans only act as incidental hosts (not necessarily maintaining parasite circulation in a population) [13],[14]. To be considered as an effective reservoir a mammal species must (i) have a high degree of exposure to sand fly vectors (as a primary blood-feeding source), (ii) be able to host the parasite for long periods without developing the disease, and (iii) be known to have been infected with parasite strains implicated in human cases [15]–[17].

In tropical America, transmission of leishmaniasis is believed to have traditionally been restricted to humid sylvatic habitats in which humans were exposed to the parasite during forest-related activities [14],[18],[19]. However, human-induced habitat transformation has facilitated rapid invasion of some vector and mammal species into non-sylvatic habitats thereby increasing both human exposure and risk of infection [8]. The dynamics of the disease are correlated with population fluctuations in reservoirs and vectors [20],[21], and strongly correlated with environmental changes [18] and climatic factors [14],[22]. Because climatic factors can lead to species' range shifts, analyses of vector and reservoir species' distributional responses to climate change scenarios provide insight into how the spatial epidemiology of leishmaniasis may be affected by climate change [23]. In particular, estimating the potential future distributions of vector and reservoir species can help identify potential risk areas for human infection.

Ecological niche models (ENMs) based on point occurrence data, digitized environmental layers, and machine learning algorithms, typically all overlaid on a Geographic Information System (GIS) platform, provide a useful framework for understanding the geography of vector-borne diseases [19], [24]–[26]. Ecological niche modeling is based on attempting to predict the fundamental niche of a species which is defined as the set of biotic and abiotic environmental conditions in which it can maintain populations without immigrational subsidy [27]. When projected to geographical space, the fundamental niche gives the potential distribution of a species. Constraints on dispersal due to geography, as well as negative ecological interactions (such as competition), may prevent a species from occupying the entirety of its fundamental niche [28]–[30]. Taking such factors into account generates the actual geographical distribution of a species. In practice, ENMs incorporate both the ecological requirements and spatial locations of species and predict species occurrences in an area between the potential and actual distributions. These distributions then have to be modified using the constraining factors mentioned above to obtain the actual distributions. ENMs are thus useful for providing a framework to test hypotheses regarding the role of different environmental variables in determining species' distributional patterns [25], [31]–[33].

For leishmaniasis in North America, *L. m. mexicana* is responsible for most human cutaneous cases of leishmaniasis and has been isolated from diverse mammal reservoir and sand fly vector species in México [34]–[36] and the United States [37]–[40]. Along the México—United States border, the cutaneous form of the disease occurs in semi-arid scrubland habitats of the Sonoran and Tamaulipan biotic provinces [37],[38], in which the sand fly species, *Lutzomyia diabolica* and *Lutzomyia anthophora*, are the presumed vectors [39]. In this region, the parasite has been isolated from the white-throated and southern plains woodrats, *Neotoma albigula* and *Neotoma micropus*; to the east it is also found in the eastern woodrat, *Neotoma floridana* [21],[37],[38]. Transmission has been observed to be restricted to localized areas, with highest prevalence in autumn [21],[37],[38],[41]. We also included the Mexican woodrat, *Neotoma mexicana*, in this study as a potential reservoir because it shows wide geographic overlap with *Lutzomyia diabolica* and *Lutzomyia anthophora* and has been incriminated as a reservoir for *Trypanosoma cruzi* [42]. It is thus likely to be a competent reservoir for *L. m. mexicana* because Trypanosoma and Lutzomyia are both kinetoplastid protozoa and are thus likely to share some of the same reservoirs.

In north México, disease cases were reported in the north, between 1986 and 1999, in the states of Tamaulipas, Nuevo León, Coahuila, and Chihuahua [43]. In the United States, Leishmania parasites have been isolated in Texas, Arizona, Oklahoma, and Ohio from humans, dogs, rodents, and insects [21],[37],[38],[41]. The (human) disease is autochthonous in Texas; mucocutaneous, localized cutaneous, and diffuse leishmaniasis have been reported [44]–[47]. By late 2009, 40 cases of leishmaniasis had been reported in Texas [46] [Chad McHugh, personal communication], and two cases had been reported in Oklahoma [Kristy Bradley, personal communication].

It is likely that the occurrence of human cases is strongly correlated with the presence of competent vector and reservoir species in sufficiently high densities [20],[21]. In this study, we begin by constructing ENMs for these species to predict their potential geographical distributions. We then project models showing sufficient predicitive power to future climate scenarios for 2020, 2050, and 2080. We use the A2 and B2 scenarios for which interpolated climate layers based on the 2003 Third Intergovernmental Panel on Climate Change (IPCC) Assessment Report [48] are publicly available. Except for the A1FI scenario, which is unlikely because it assumes no carbon emissions reduction, the A2 scenario is the most extreme of the six canonical IPCC emissions scenario [49]; the B1 scenario is the most conservative but, since interpolated climate layers were not available for it, we used the related B2 scenario which is also conservative. We assumed that results invariant under both scenarios are likely to be robust.

A variety of methods have been proposed for the assessment of disease risk though a standard framework is yet to emerge [50]–[53]. Here, disease risk was assessed using only two risk components. The first was simply the potential for the presence of both vector and reservoir species based on (i) the quality of available habitat as predicted by the ENMs and (ii) dispersal ability, that is, patterns of dispersal from their ranges at the previous time that was modeled. The second was the projected “cost” measured by the number of people potentially exposed to the disease. Different projected future population estimates were used for the A2 and B2 scenarios. These are not the only components of disease risk. For the spread of leishmaniasis, three categories of risk have traditionally been distinguished [8], those due to: (i) anthropogenic transformations and other environmental changes; (ii) immunological profiles of human populations; and (iii) treatment failure and drug resistance. Leishmaniasis has also been associated with poverty in one recent analysis [54] which would affect factors in all three categories; it is known to be less prevalent in urban settings than rural contexts [53]. This analysis is restricted to only one element of risk from environmental changes: that due to climate change, which will interact with other risk factors in complex ways which are beyond the scope of this study. However, this element of risk, the “ecological risk,” provides an epidemiological baseline for consideration of other aspects of risk. If the ecological risk is low, then other risk factors will typically not matter very much: if disease vectors and reservoirs cannot survive in an area because of ecological reasons, then there is little likelihood that the diseases can become autochthonous in that region. If the ecological risk is high, the other factors will be critical to the control of the disease. In what follows, for expository brevity, we will use “risk” to refer only to this ecological risk of leishmaniasis spread.

## Methods

### Study area

The study area for model construction consisted of all the terrestrial regions of Canada, the United States, and México delimited by the 14.13° N line of latitude to the south at the México—Guatemala border. It was divided into 41 680 234 cells (average area: 0.50 sq km [SD = 0.33]) at a resolution of 30 arc-seconds (0.00833°) of latitude and longitude. All of continental Canada and the United States were included in order to identify all areas in which potential distributional shifts of vector and reservoir species would place them at risk for leishmaniasis through a northward range expansion of the disease.

### Vector and reservoir species records

The two known Lutzomyia vector species found in this region and known to have epidemiological relevance, *Lutzomyia anthophora* and *Lutzomyia diabolica*, were included. For reservoirs, we included all Neotoma species widely distributed in northern México or the southern United States. *Neotoma albigula*, *Neotoma micropus*, and *Neotoma floridana* are known reservoirs; *Neotoma mexicana* is believed to be a reservoir [26].

**Figure 1:**
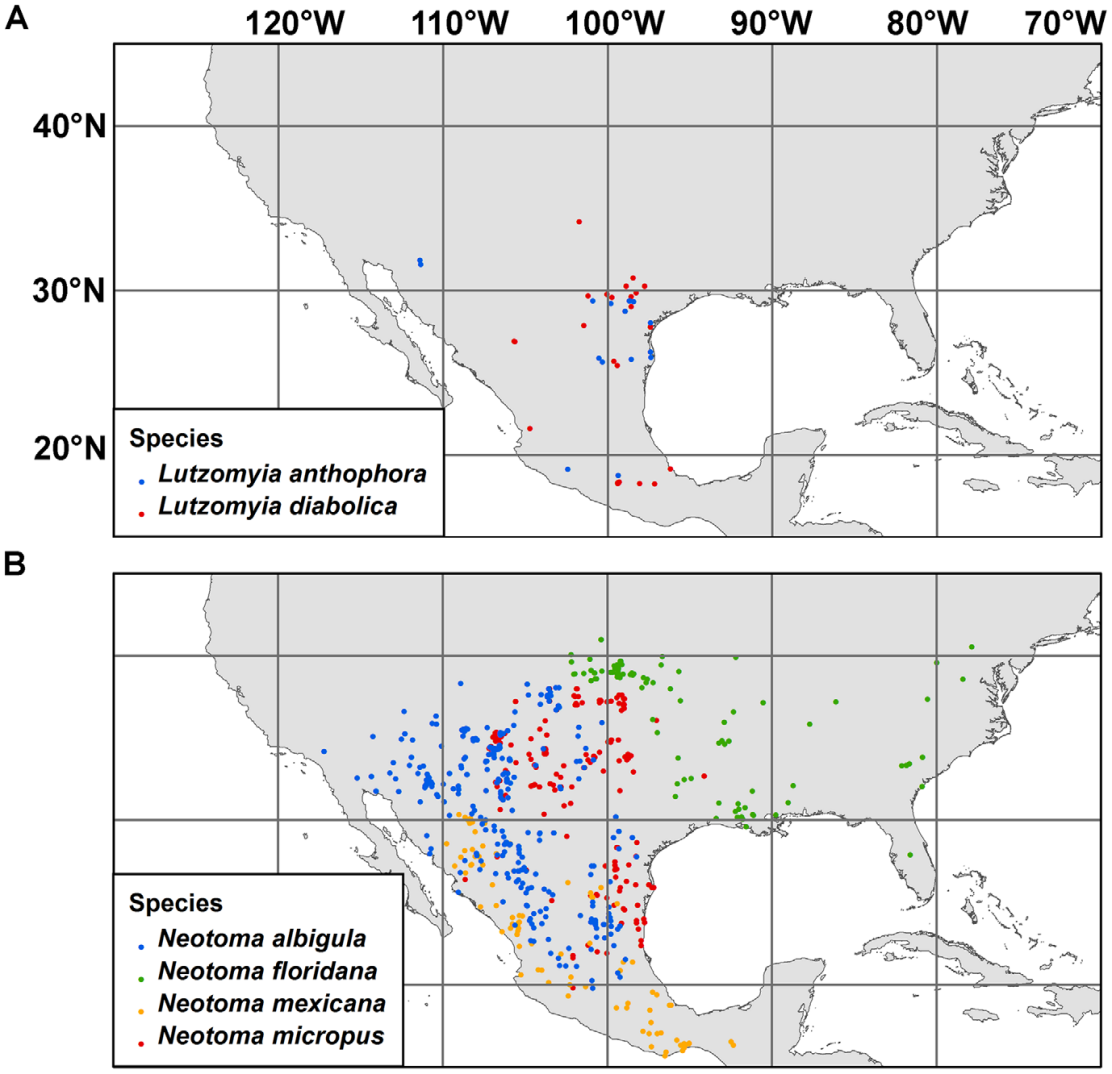
Vector and reservoir data points in North America. (a) Both vector species are shown. (b) All four reservoir species are shown.

For ENM construction, species point occurrences were obtained from the Disease Vectors Database (www.diseasevectors.org; last accessed 24-February-2009) which provides free publicly-accessible data on both vector and reservoir occurrence records [55]. Reservoir point locality records were obtained from museum mammal collections (all of which are listed in the Acknowledgments). Vector point locality records were obtained from published literature [40],[56],[57], and personal communications (Chad McHugh, 2007), all included in the Disease Vectors Database [55]. Figure 1 shows the reservoir and vector species occurrence points used for ENM construction. Because of the fine resolution of the study, and in order to ensure concordance between species' records and the environmental layers used, only post-1990 occurrence points were used in model construction. Seventeen occurrence points were available for *Lutzomyia anthopora*, 31 for *Lutzomyia diabolica*, and 1047, 192, 103, and 574 for *Neotoma albigula*, *Neotoma floridana*, *Neotoma mexicana*, and *Neotoma micropus*, respectively.

For three of the reservoir species, *Neotoma micropus*, *Neotoma floridana*, and *Neotoma mexicana*, an independently-derived data set was used to test the ENMs, with 31, 13 and 28 records available for the three species (respectively). This data set was obtained from US mammal collections listed below (see Acknowledgments). None of these data were used either in model construction or for the internal model validation (through the training and testing process in Maxent). For *Neotoma albigula*, these data were not available as most specimens deposited in Mexican mammal collections are currently under taxonomic revision. Nonetheless, a high number of point localities were used in model construction resulting in a reasonable robust ENM for this species (see Results).

### Environmental layers and climate projections

Nineteen bioclimatic data layers were used as explanatory variables in the ENMs (see Table 1). For the present climate, the data were obtained from the WorldClim database (www.worldclim.org; last accessed 15-Nov-2009) where they were available at the resolution used in this analysis [58]. (For future climate scenarios, these layers had to be computed as discussed below.) Topographical variables (elevation, slope, and aspect) were obtained from the Hydro 1k data set [59]. These 22 layers were used for ENM construction.

**Table 1:** Explanatory variables used for the construction of niche models.

Variable	Explanation
BIO1	Annual Mean Temperature
BIO2	Mean Diurnal Range (Mean of monthly [max. temp. – min. temp.])
BIO3	Isothermality
BIO4	Temperature Seasonality
BIO5	Maximum Temperature of Warmest Month
BIO6	Minimum Temperature of Coldest Month
BIO7	Temperature Annual Range
BIO8	Mean Temperature of Wettest Quarter
BIO9	Mean Temperature of Driest Quarter
BIO10	Mean Temperature of Warmest Quarter
BIO11	Mean Temperature of Coldest Quarter
BIO12	Annual Precipitation
BIO13	Precipitation of Wettest Month
BIO14	Precipitation of Driest Month
BIO15	Precipitation Seasonality
BIO16	Precipitation of Wettest Quarter
BIO17	Precipitation of Driest Quarter
BIO18	Precipitation of Warmest Quarter
BIO19	Precipitation of Coldest Quarter
	Elevation
	Slope
	Aspect

For details of the computation of these parameters from a basic set [BIO5, BIO6, BIO13, BIO14], see the WorldClim web-site [www.worldclim.org].

For the future climate projections, monthly values for maximum and minimum temperature and precipitation were available at the WorldClim database at the resolution used in this analysis. For the A2 scenario, we used the CSIRO model because it predicts the highest temperature increase, for the B2 scenario, we used the Hadley model which predicts the lowest temperature increase. These layers had been interpolated from the Third IPCC Assessment Report. From these layers, the 19 bioclimatic variables used for ENM construction (Table 1) were computed using an ArcInfo AML script (mkBCvars.aml Ver 2.3) also provided at the WorldClim database.

### Ecological niche models

ENMs were constructed using the Maxent software package (Version 3.2 [60]–[62]). Maxent has been shown to be robust for ENM construction from presence-only data [31]. Maxent allows predictive models based on current climatic and other environmental data to be fitted to future climate projections. The species and environmental data have already been described.

Following published recommendations, Maxent was run without the threshold feature or duplicates so that there was at most one sample per pixel; linear, quadratic, and product features were enabled; 75% of the data were used to construct the models and 25% were used to test them [63]. The convergence threshold was set to a conservative value of 1.0×10^−5^ [61]–[63]. The accuracy of each model was assessed using three tests:

1. The AUC (area under the receiver operating characteristic [ROC] curve) was calculated for each model using the proportion of the study area in which the species is predicted to be present. This is automatically generated by Maxent which constructs ROC curves using randomly selected pseudoabsences. For acceptable models, the AUC threshold was set to an extreme conservative value of 0.95 for both training and test data.
2. Eleven binomial tests of model performance [24],[63] which are reported as part of Maxent output were performed on the data. All eleven binomial tests were required to be significant at *p*<0.01 which is also a conservative choice.
3. Model predictions were compared with the independently derived data set of species' occurrences for the three species for which these data were available (*Neotoma micropus*, *Neotoma floridana*, and *Neotoma mexicana*). First, all predicted cells with probability<0.01 were dropped from the potential habitat of a species. Next, from the remaining cells, the top 50% of the cells were retained; as explained below, these correspond to the middle threshold choice for the distribution of a species, as explained in the next paragraph. We then calculated how many of the new occurrence points for each species fell within the predicted distribution. To obtain the statistical significance of this result, we compared this number to that which is obtained if 10 000 sets of points are randomly drawn from an area. However, this parameter is sensitive to the area from which the random points are drawn. If it is sufficiently large, we would get spurious significance results. To avoid this problem we drew the points from the smallest box, bounded by longitude and latitude lines, that included all the occurrence points. However, this test does not address the likely problem that the occurrence data were probably not collected using a randomized survey procedure. However, for rodents in México and Texas, which are the regions from which the new occurrence data are available, collection efforts have been fairly extensive and most areas are likely to have been sampled.

The second and third tests were used because the AUC alone may lead to misplaced confidence in an ENM [64],[65]. Maxent models were first developed from all the topographic variables and the bioclimatic variables from 2003. These models were projected to the climate scenarios for 2020, 2050, and 2080 with the same topographic variables.

The logistic output from Maxent consists of the predicted probability of occurrence for each species in each cell. These probabilities represent the potential distribution of a species. The next step is to predict actual geographical distributions. In this analysis, the probabilities were converted to geographical distributions using three different thresholds. First, all cells with a predicted probability<0.01 were dropped from the potential habitat of a species. Next, from the remaining cells, the top 10, 50, and 90% of the cells were retained for the 10, 50, and 90 percentile models. Thus, the 10 percentile model is the most conservative, the 50 percentile model is less so, and the 90 percentile model is the least conservative about the occurrence of a species. Finally, for both the A2 and B2 scenarios, using these percentile models for the species, we constructed models consisting of areas in which at least one vector and one reservoir is present at the 10, 50 and 90 percentile levels.

### Species dispersal

In general, environmental factors may influence species' spread to new habitat. Range shifts in response to climate change have now been empirically documented for a wide range of species [66]. However, a variety of contingent factors including dispersal ability, geographical barriers, and negative interactions with other species may prevent species from occupying the entirety of their environmentally suitable habitats [28]. When these complexities are taken into account, range shifts of species across landscapes remain poorly theoretically understood though models of dispersal have begun to receive the attention that they deserve [67].

Given that there is no dispersal model available for any of the leishmaniasis vector or reservoir species, two extreme models were used here: (i) the *universal* dispersal model assumed that each species occupied all of its suitable habitat, that is, there is no limit to dispersal beyond environmental suitability; and (ii) the *contiguous* dispersal model assumed that a species occupied a suitable cell only if it was connected to the range of the species at the last temporal stage through a pathway of suitable cells. Thus, between 2000 and 2020 the dispersal of a species is restricted only to those cells that are environmentally suitable and adjacent to an occupied cell in 2000. The same pattern is repeated for future time steps. Given the resolution of the analysis, this means that the species can at least disperse over a distance of about 1 km. over two decades (which is conservative).

### Human population projections for risk assessment

Human population data for the year 2005 and projections for the years 2020, 2050, and 2080 under the A2 and B2 climate change scenarios were obtained from the Global 0.5-deg Gridded Population Dataset (www.sjziam.ac.cn/sjziam/kyxt/shenyj.htm; last accessed 01-April-2009; [68]) based on the IPCC Special Report on Emissions Scenarios [49]. The distribution layers had a resolution of 0.5° and were resampled to the resolution of this analysis using ArcMap 9.2 maintaining the same population density as in the original projections. These layers were overlaid with the models that predicted the presence of at least one vector and one reservoir species. The human population potentially exposed to leishmaniasis was computed as that of those cells in which at least one vector and reservoir species was present at the 10, 50, and 90 percentile levels.

## Results

### Model output

**Figure 2:**
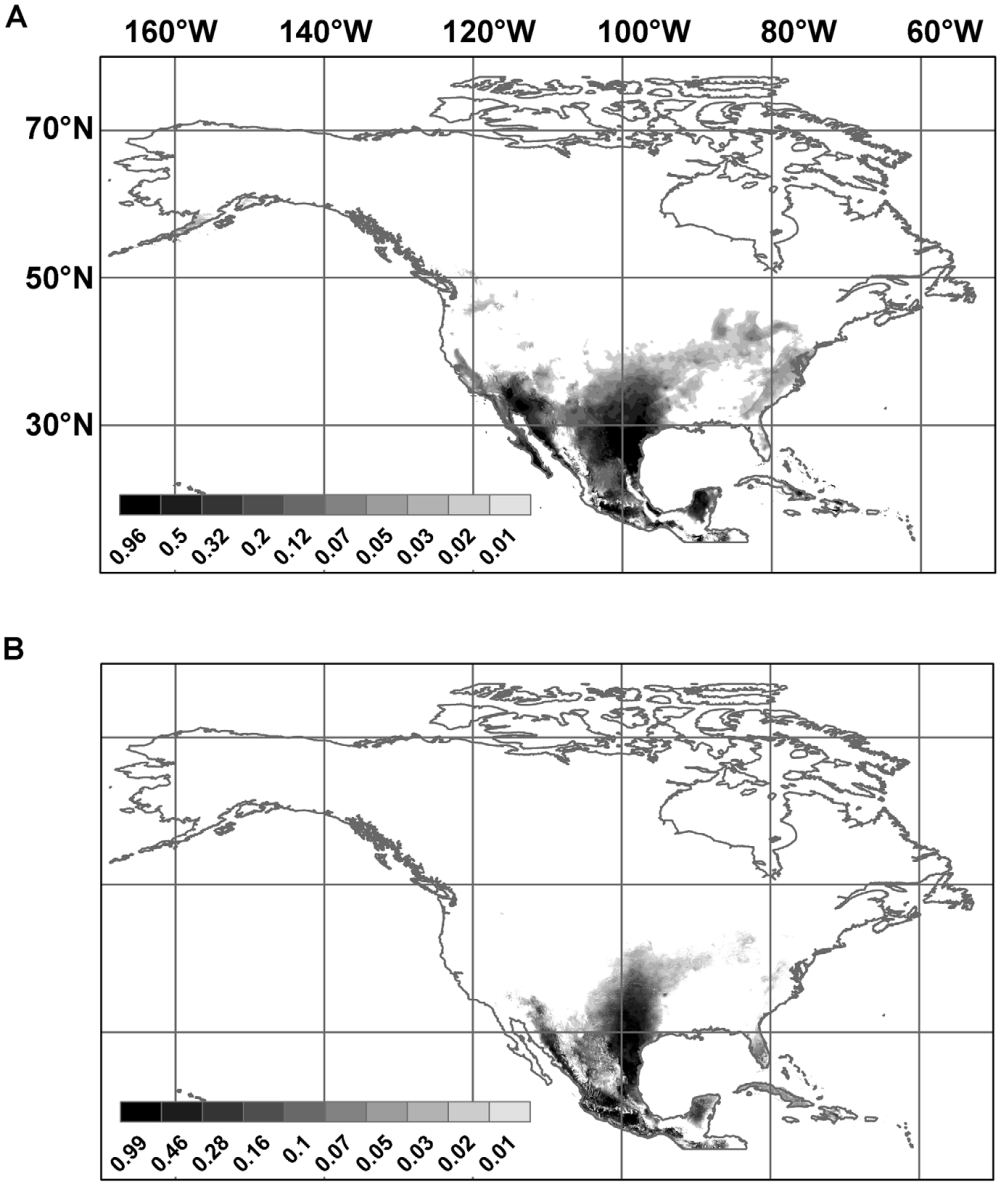
Predicted current distributions for leishmaniasis vector species. The figures show the geographical projection of the ecological niche model. (**a**) *Lutzomyia anthophora*; (**b**) *Lutzomyia diabolica*.

**Figure 3:**
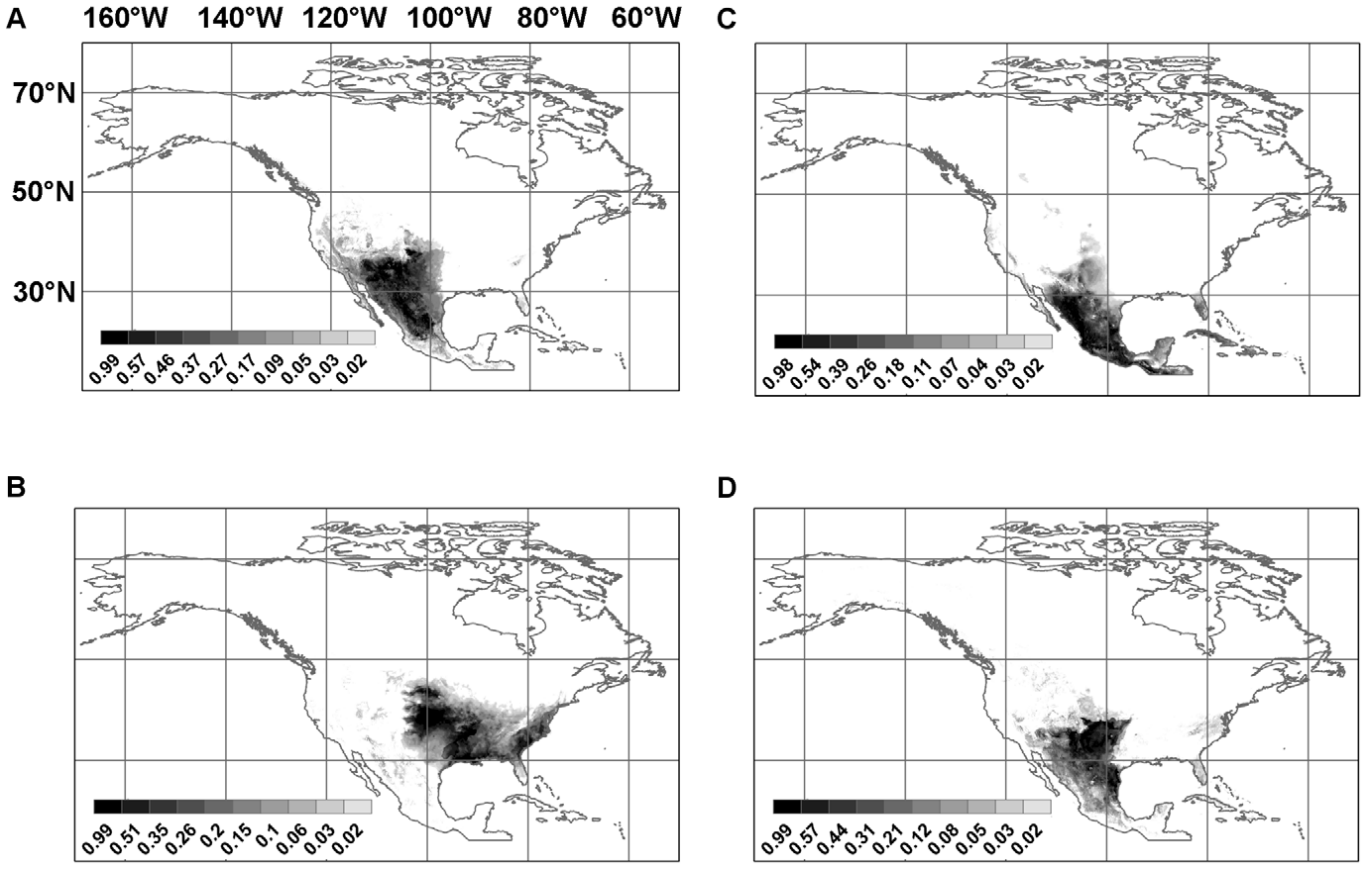
Predicted current distributions for leishmaniasis reservoir species. The figures show the geographical projection of the ecological niche model. (**a**) *Neotoma albigula*; (**b**) *Neotoma floridana*; (**c**) *Neotoma mexicana*; (**d**) *Neotoma micropus*.

Figure 2 shows the present predicted distributions of *Lutzomyia anthophora* (2a) and *Lutzomyia diabolica* (2b). Figure 3 does the same for *Neotoma albigula* (3a), *Neotoma floridana* (3b), *Neotoma mexicana* (3c), and *Neotoma micropus* (3d). The AUCs ranged from 0.963 to 0.984 for both training and test data; these values are above our conservative threshold. For all six species, the 11 *p*-values for Maxent's internal binomial tests were all less than 0.01. When tested against the independently-derived data set, for *Neotoma floridana*, 0 out of 13 points fell outside the predicted range (*p*<0.0001), for *Neotoma mexicana*, 1 out of 28 points fell outside (*p*<0.0001), and for *Neotoma micropus*, 2 out of 31 points fell outside (*p*<0.0001).

The topographic parameters were not explanatorily significant (data not shown). There was no obvious pattern with respect to the climatic parameters. For *Lutzomyia anthophora* the two most important parameters were the mean temperatures of the wettest and warmest quarters, for *Lutzomyia diabolica* they were the annual mean temperature and the minimum temperature of the coldest month, for *Neotoma albigula*, isothermality and mean diurnal temperature range, for *Neotoma floridana*, the maximum temperature of the warmest month and the minimum temperature of the coldest month, for *Neotoma mexicana*, isothermality and precipitation seasonality, and for *Neotoma micropus* the mean temperatures of the wettest and driest quarters.

**Figure 4:**
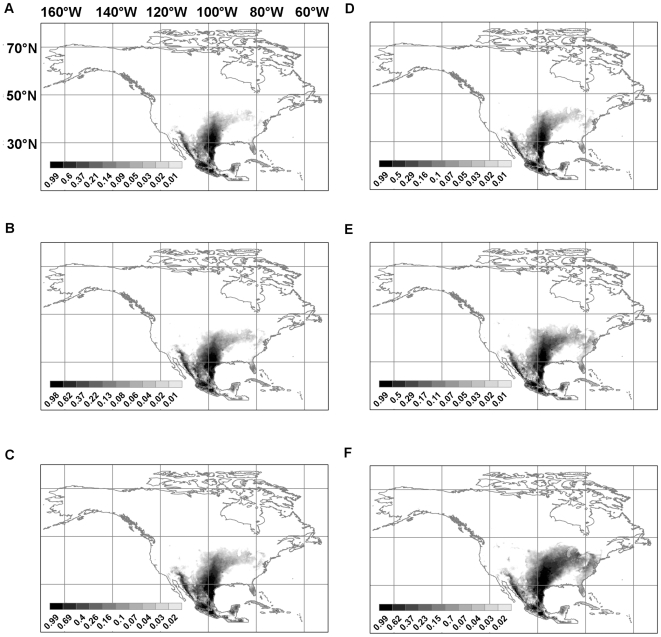
Predicted future distributions for *Lutzomyia diabolica*. (**a**) B2 scenario, Hadley model, 2020; (**b**) B2 scenario, Hadley model, 2050; (**c**) B2 scenario, Hadley model, 2080; (**d**) A2 scenario, CSIRO model, 2020; (**e**) A2 scenario, CSIRO model, 2050; (**f**) A2 scenario, CSIRO model, 2080.

**Figure 5:**
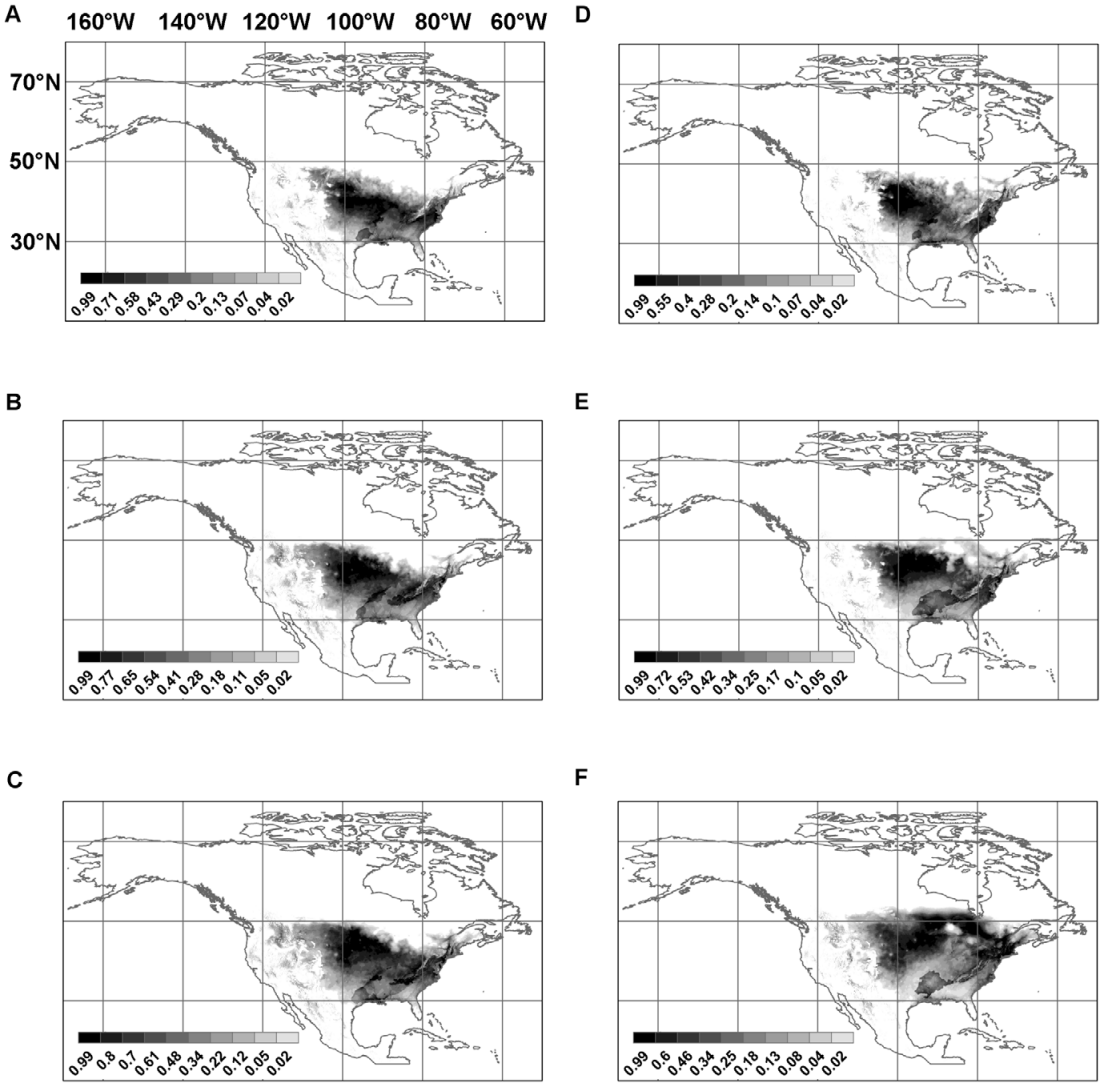
Predicted future distributions for *Neotoma floridana*. (**a**) B2 scenario, Hadley model, 2020; (**b**) B2 scenario, Hadley model, 2050; (**c**) B2 scenario, Hadley model, 2080; (**d**) A2 scenario, CSIRO model, 2020; (**e**) A2 scenario, CSIRO model, 2050; (**f**) A2 scenario, CSIRO model, 2080.

Figure 4 shows the future predicted distributions for *Lutzomyia diabolica* in 2020, 2050, and 2080 under both the B2 (Hadley model) and A2 (CSIRO model) future climate scenarios. Figure 5 does the same for *Neotoma floridana*. These two species were chosen for presentation here because, on average, they show the largest range expansions that have the most relevance for the potential spread of leishmaniasis northwards. Results for the other four species (*Lutzomyia anthophora*, *Neotoma albigula*, *Neotoma mexicana*, and *Neotoma micropus*) are available in Figures S1, S2, S3, and S4.

### Predicted distribution changes

Table 2 records the percent change in area of a species' range from one time stage to the next for the universal and contiguous dispersal models for the A2 and B2 climate change scenarios. The last three columns report the same numbers for the area occupied by at least one vector and one reservoir species. If we assume that each vector and reservoir is competent for human transmission, and that both vectors are associated with all four reservoirs, then these numbers are the most relevant ones for the risk of spread of leishmaniasis. As expected, area changes are much larger for the universal model than for the contiguous model.

**Table 2:** Shift in distribution area of species.

		*Lu. anthophora*			*Lu. diabolica*			*N. albigula*			*N. floridana*			*N. mexicana*			*N. micropus*			At least one vector and reservoir		
		20	50	80	20	50	80	20	50	80	20	50	80	20	50	80	20	50	80	20	50	80
10	A2	20.8	27.6	34.7	−5.2	31.8	36.1	15	8.7	25.4	13.6	17.5	23.5	−11.8	0.9	11	−1.9	13.4	27.7	28.4	19.1	21.6
		19.4	26.6	33.5	−6.6	29.6	36.9	8.6	9.2	23.7	7.2	19.1	−6	−14.4	1.1	6	−9.6	15.1	23.2	22.4	8.8	34.9
	B2	20.1	1.9	7.7	−1.1	16.4	8.1	9.5	10.7	13.8	14.4	18.3	10.1	−19	10.1	−4.3	−6.4	18.2	5.2	18.9	8.6	12.1
		17.4	4.2	7.7	−2.4	15.3	9.4	2.9	11.4	15.8	14.4	28.1	13.9	−21.8	10.7	−6.5	−13.5	12.4	16.2	13.8	9.4	8.9
50	A2	21.5	25.9	28.2	−0.6	29.2	34.1	15.6	11.2	8.5	18.5	20.1	24	−8.9	1.5	7.7	0.1	16	23.6	22.1	24.3	17
		19.2	26.1	25.6	−2	27.2	35.3	14.1	10.7	8.6	15.7	22	23.5	−9.2	1.3	7.7	−1.9	16.1	23.6	22.1	23.3	16.9
	B2	19	3.5	4.6	2.3	16.8	7.7	13.8	10.2	11.2	19.3	16.7	12.8	−16.8	10.2	−5.4	−1.8	17	3.6	17.6	7.4	7.8
		18.2	3.4	5.1	1.6	15.45	8.7	11.9	10.7	10.7	16.3	18.7	13	−17.2	9.9	−5.4	−3	16.9	3.2	15.2	7.9	8.4
90	A2	8.8	27.8	21.5	27.1	19	23.2	21.8	12.6	21	24.6	19.6	26.5	−1.4	2.1	5.8	11.3	13	20.8	20.9	22.8	13.8
		8.1	23.8	17.6	24	20.6	23.5	19.9	13.5	20.6	23.9	19.6	26.1	−1.4	2.2	5.5	9.6	12.5	20.2	19.8	22.3	14.1
	B2	13.9	−1.2	4.8	15.2	15	9.3	18.8	11.1	9.4	24.1	17.8	10.5	−7.5	8.8	−6.7	11.5	11.2	4.9	17.4	7.4	5.3
		13.1	−1	4.7	13.6	15.3	9.6	17.7	13.5	8.9	22.6	18.4	10.6	−7.5	8.6	−6.5	9.7	11.2	4.9	16.6	7.3	5.6

Each cell records the percent change in the area of a species' range from one time stage to the next (upper entry: universal model; lower entry: contiguous model). The first column is the threshold percentile and the second is the climate scenario (A2: CSIRO; B2: Hadley).

**Figure 6:**
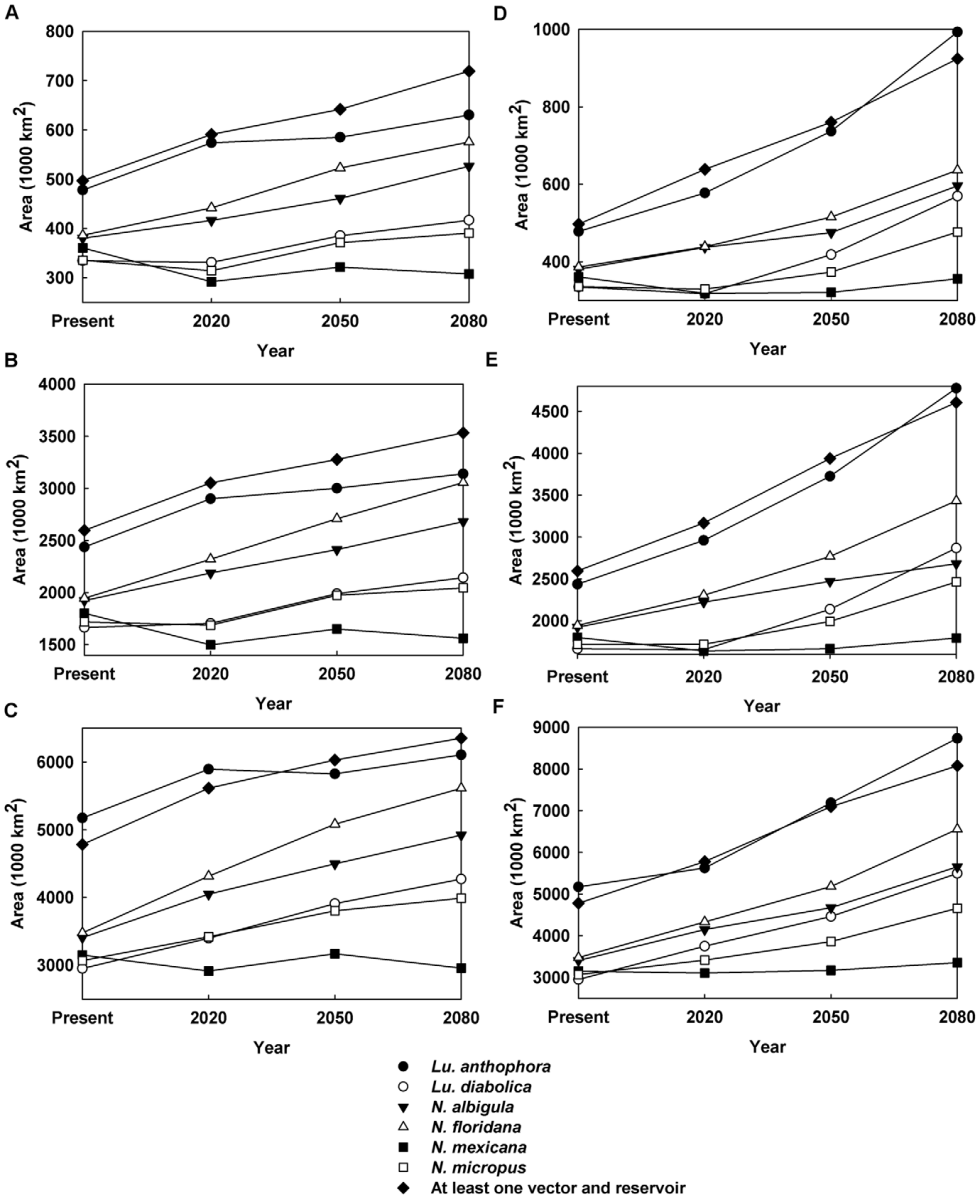
Range expansion of vector and reservoir species under the universal dispersal model. (**a**) B2 scenario, Hadley model, top 10% of the habitat; (**b**) B2 scenario, Hadley model, top 50% of the habitat; (**c**) B2 scenario, Hadley model, top 90% of the habitat; (**d**) A2 scenario, CSIRO model, top 10% of the habitat; (**e**) A2 scenario, CSIRO model, top 50% of the habitat; (**f**) A2 scenario, CSIRO model, top 90% of the habitat.

**Figure 7:**
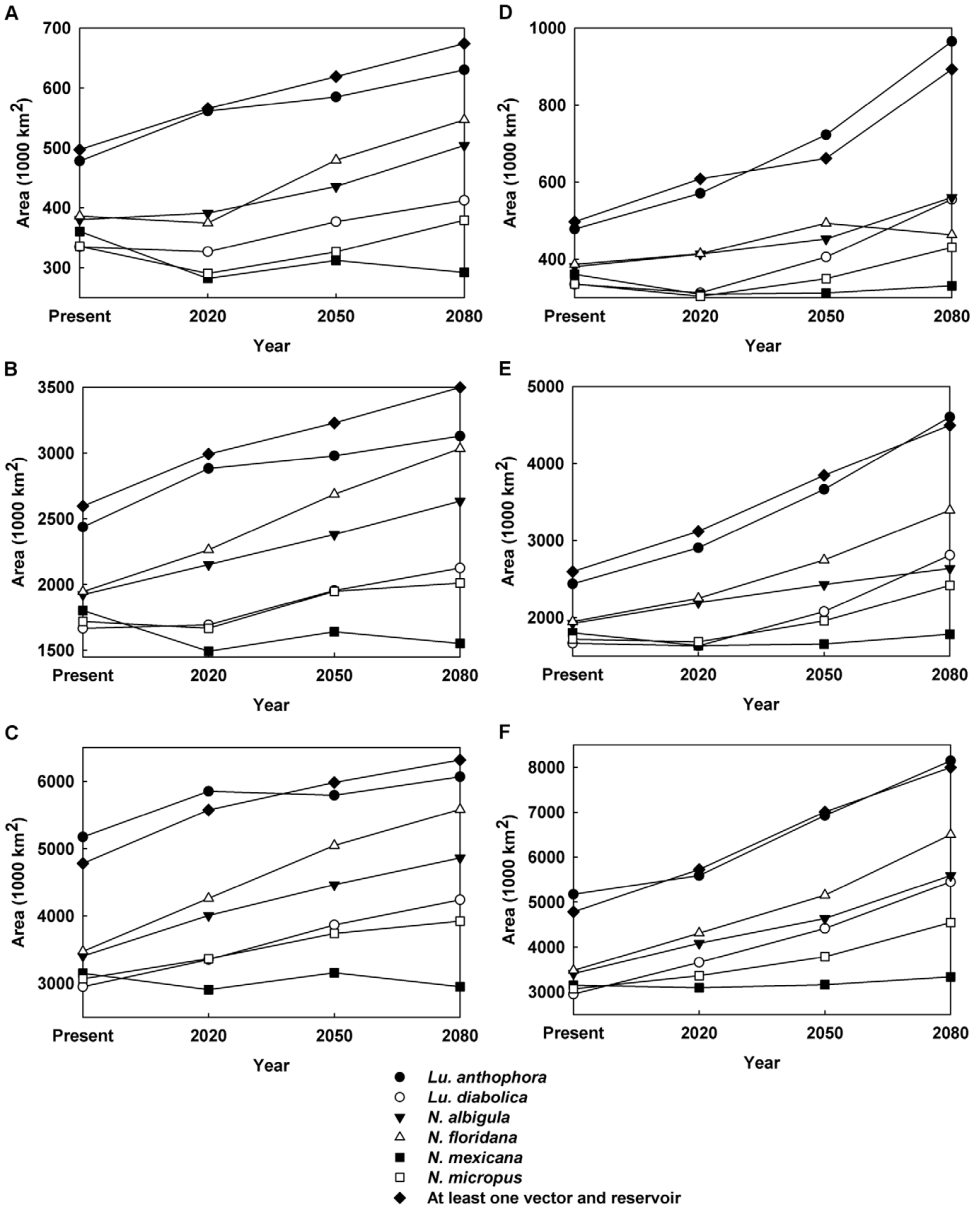
Range expansion of vector and reservoir species under the contiguous dispersal model. (**a**) B2 scenario, Hadley model, top 10% of the habitat; (**b**) B2 scenario, Hadley model, top 50% of the habitat; (**c**) B2 scenario, Hadley model, top 90% of the habitat; (**d**) A2 scenario, CSIRO model, top 10% of the habitat; (**e**) A2 scenario, CSIRO model, top 50% of the habitat; (**f**) A2 scenario, CSIRO model, top 90% of the habitat.

The change in total range is plotted in Figure 6 for the universal dispersal model and in Figure 7 for the contiguous model. For both dispersal models, for the (conservative) B2 climate change scenario, the predicted range of *Neotoma mexicana* ultimately contracts by 2080 irrespective of which quality of habitat (the top 10, 50, or 90 percentile ranges) is deemed to be occupied; under the (extreme) A2 scenario it increases only slightly after a decrease in 2020 except for the highest quality habitat (Figure 7d) which decreases. Otherwise predicted range expansions are ubiquitous though, in many cases, there is an initial decrease in 2020 only to be followed by rapid increase in 2050 and 2080. Though large range expansions are seen for *Lutzomyia anthophora*, much of this is not in the northerly direction which would increase the geographical range of leishmaniasis. If we consider areas in which at least one vector and one reservoir species are present and, therefore, there is potential human exposure to leishmaniasis, the range always expands and, as expected, the expansion is greater for the A2 scenario than the B2 scenario. In the latter case, there is no visible difference between the contiguous and universal dispersal models in the case for all habitat classes (Figures 6d -f, 7d -f) and the range is expected to expand by as much as 60%.

### Potential human impacts

**Figure 8:**
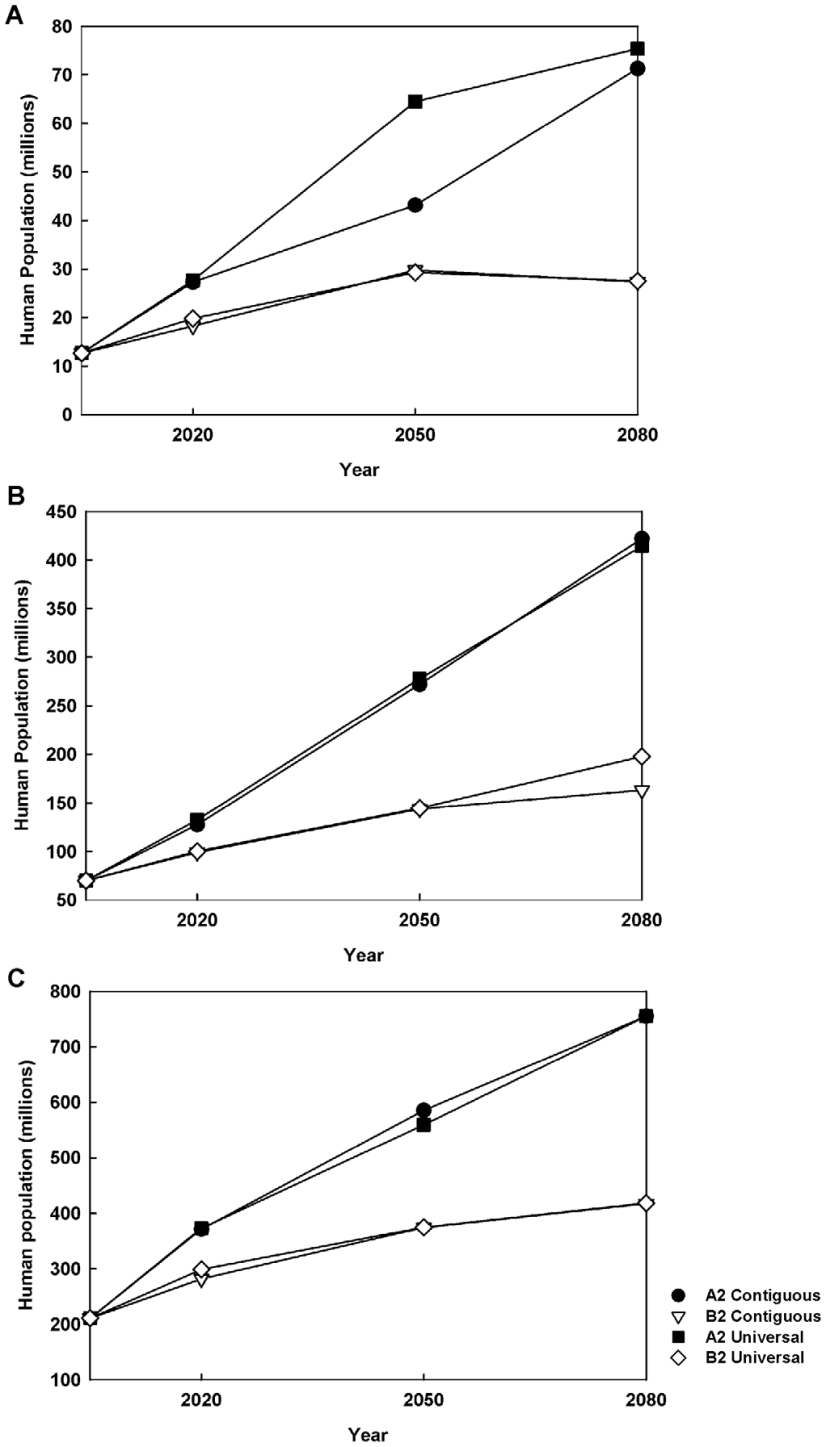
Human population risk due to the presence of at least one vector and reservoir species. (**a**) Top 10% of the habitat; (**b**) Top 50% of the habitat; (**c**) Top 90% of the habitat.

Figure 8 records the potential human exposure in terms of the number of individuals who would be present in a cell in which at least one vector species and one reservoir species is expected to be present. This number is uniformly larger for the A2 scenario than the B2 scenario. If we restrict attention to the population in only the best habitat (the 10 percentile case), for the A2 scenario there is some difference between the contiguous and universal dispersal models with the latter, as expected, leading to more exposure. Otherwise the results are remarkably robust with respect to variation in dispersal behavior. The best case scenario is the one in which both the vector and reservoir species are restricted to the top 10% of their predicted habitat and climate changes according to the B2 scenario (Figure 8a, lower two graphs). Even though the range of the disease is predicted to decline after 2050, when it peaks, the value in 2080 is about 27×10^6^ individuals, which is more than twice the current value of 12×10^6^ individuals.

## Discussion

There is a high potential for the spread of leishmaniasis in North America, north of México and Texas, because of climate change. The main reasons for this possibility are range shifts for *Neotoma floridana* and *Lutzomyia diabolica* in eastern North America, and for *Neotoma micropus* and *Lutzomyia anthophora* further west. Figure 5 shows the predicted distribution of *Neotoma floridana* in 2020, 2050, and 2080. Under both climate scenarios its range expands northwards and more so under the A2 scenario than the B2 scenario. The same pattern is predicted for *Lutzomyia diabolica* but to a lesser extent (Figure 4). Though the predicted range expansion of *Lutzomyia anthophora* is more extensive than that of *Lutzomyia diabolica*, the northward shift is not as pronounced. It is, therefore, likely that the risk of leishmaniasis spread in eastern North America will be limited by the range expansion of *Lutzomyia diabolica*. Much of the eastern half of the United States and, under the A2 scenario, a part of southeast Canada is at risk. The southern boundary of the range of *Neotoma floridana* also incrementally shifts northwards. This will make some of the southern extremities of its present range unsuitable in the future. However, if *L. m. mexicana* is already established within this species—as suggested by the evidence from Texas (see below)—and expanding its range, it will move north with *Neotoma floridana*.

For the central United States, the predicted increased risk comes from the northward expansion of *Lutzomyia anthophora* and *Neotoma micropus* though this risk does not extend into Canada. While there is potential for range expansion of *Neotoma micropus* into Canada, the risk of leishmaniasis will be limited by the northern boundary of the range of *Lutzomyia anthophora* (see Figure S1 and Figure S2 of Supplementary Materials). In the western United States the predicted increased risk is due to the same vector and range expansion of *Neotoma albigula* (see Figure S3). Once again, it may be limited by the range of *Lutzomyia anthophora*. *Neotoma mexicana* is not predicted to play any significant role.

However, factors not taken into account here may impede the spread of leishmaniasis to the west. Mead and Cupp [69] found an association of *Lutzomyia anthophora* and *Neotoma albigula* in Pima County, Arizona, which is in accordance of our predictions (Figures 2a and 3a). Subsequently Kerr et al. [38] confirmed the presence there of *L. m. mexicana* in *Neotoma albigula* individuals. However, this is the western limit of known leishmaniasis foci in the United States and occurs in a riparian zone in the Sonoran biotic province. If precipitation and water availability has been preventing the spread of the disease further west, this impediment may be relaxed with climate change. However, at the coarse resolution used in this study, precipitation-related variables were not the ones found to be most important in the ENMs for these two species. It is possible that other features of the habitat (including vegetation composition and structure, soil type, and presence or absence of water bodies) may prevent sufficiently high densities of vectors and reservoirs in this region for disease transmission.

The reliability of these predictions depends on how successful the ENMs are. In general, ENM predictions have been known to correct traditional range maps based on marginal records and expert judgments of appropriate contiguity and habitat suitability [25]. ENM predictions for mammal species have been successfully tested in México [70]. In this analysis, for three reservoir species (*Neotoma floridana*, *Neotoma mexicana*, and *Neotoma micropus*), the models showed high concordance with an independent data set and there are grounds for confidence. For *Neotoma albigula*, and for the two vector species, the internal tests within Maxent gave good results but data were not available for independent tests.

For Texas, the predicted potential distributions for both *Neotoma floridana* and *Neotoma micropus* (see Figures 3b and 3d) are more conservative than those found in traditional range maps [71]. In central Texas, according to our predicted distributions, there is a north-south band of habitat that is at best marginally suitable for any of the four Neotoma species modeled here. This may explain the temporal pattern in the spread of leishmaniasis cases in Texas. Leishmaniasis was recorded in 1903 at the southeastern tip of Texas at the border with México [20]. By the mid-1940s, it had spread to south-central Texas, by the early 1980s, it had spread to central Texas, and by the 1990s, to north Texas. However, throughout this period it did not spread east of Gonzales County (97.51° W) even though there was suitable habitat for both vector species according to our ecological niche models. What seems to have restricted this eastward spread is the presence of at best marginal habitat for any of the Neotoma reservoir species along the north-south strip mentioned earlier. The pre-2000 records of leishmaniasis from Texas fall within the area predicted to be suitable habitat for *Neotoma micropus*.

By 2000, however, the disease had breached this barrier of unsuitable reservoir habitat in east-central Texas, and at least ten cases of leishmaniasis have been reported since, further north and east of the barrier [46],[47]. Because the patients reported that they had not travelled outside the respective counties in at least five years, the infections were thus presumably because of the establishment of a local transmission cycle [46]. This region has good habitat for *Neotoma floridana* (see Figure 3b). These theoretical results support the earlier conclusion of McHugh et al. [21] who recorded *L. m. mexicana* in *Neotoma floridana* east of the presumed barrier in 2001 and presumed that the parasite had established a life-cycle with this host species. If this scenario is correct, except for ecological suitability for vector species, there is little impediment left for the further eastward spread of leishmaniasis from Texas to other states. The results of this study show that climate change will exacerbate the present risk.

In this analysis, the risk of human exposure to leishmaniasis was estimated using the projected human population under the A2 and B2 climate change scenarios in cells in which at least one vector and one reservoir species were predicted to be present. Even under the contiguous dispersal model, and assuming that a species will occupy only the top 10% of its potential habitat, the expected number of individuals exposed to leishmaniasis is predicted to more than double to 27×10^6^ by 2080. Under less contiguous assumptions, this number becomes much higher. Because of the various uncertainties associated with such projections, the absolute numbers are open to question. Leishmaniasis typically affects only rural populations and much of the future population of this region will live in urbanized environments [3]. Consequently, the size of the human populations at serious risk will be much less than these absolute numbers. However, given that a large fraction of the population (76.9% in México and 81.4% in the United States [72]) already live in urban areas, the conclusion that the exposure risk will at least double is relatively robust and deserves attention from a public health perspective. Even if we assume urbanization will be 90%, and this is the percentage of non-exposure to leishmaniasis, 2.7×10^6^ individuals may be at risk in 2080 under the most conservative scenario.

Increased exposure need not lead to increased disease cases provided that adequate preventive measures are in place (beyond relying on natural immunity in human populations). For leishmaniasis, potential public health measures could include an expanded surveillance and control program beyond the northern boundary of the present range of the disease in the southern United States. Surveillance must expand northwards as the disease advances in that direction. Other components include expanded efforts at vector and reservoir control. If the conclusion that rodent reservoir distributions have controlled the spread of the disease in Texas is correct, a focus on rodent control may be attractive since many of these rodent species are also implicated as agricultural pests in much of their range.

Finally, eight limitations of this analysis should be emphasized. First, there is necessarily considerable uncertainty about the future, including the projected climate scenarios and human population changes. This analysis also does not consider the possible emergence of new vector species as the disease spreads north. Second, whereas ENMs have been successfully tested with present-day species distributions, it is an open question whether they are being successfully fitted to future climatic layers. Third, land use and land cover changes were not taken into account because they are hard to predict with much confidence. For this reason, land cover was also not used in constructing the ENMs (though it is likely that, in general, they would lead to increased accuracy of predictions). Fourth, as explicitly noted earlier, species' dispersal remains poorly understood. Though many conclusions remain robust under the two extreme dispersal models considered here, other patterns of dispersal may lead to different conclusions. Fifth, we have no definitive estimate of how much of a species' potential habitat it will occupy in the future even beyond uncertainty about dispersal. We used three percentile ranges (10, 50, and 90) and many quantitative conclusions depend on these values. The most important point is that, even under the most conservative assumptions, there is a serious risk of both the increase of the geographical range of leishmaniasis and the number of human individuals potentially exposed to it.

Sixth, as explained in the Introduction, this analysis only considers ecological risk and it remains possible that other human determinants of disease risk (for instance, public health initiatives) and natural determinants (such as regional variation in immunity) may qualify some of the conclusions arrived at here. These factors are beyond the scope of this analysis but must be acknowledged when interpreting its results. Seventh, even within the ecological context, we did not take into account differences between the vector and reservoir species, in effect assuming that each vector has the same affinity for each reservoir, and that all vectors and reservoirs are equally competent at maintaining the parasite and transferring it to humans. At present there is insufficient data for such differences to be incorporated into our models. Finally, we did not take into account the ecological factors that may directly affect the life-history of the parasite itself and just presumed that it can flourish wherever an appropriate vector-reservoir cycle is established. This is also open to question.

### Conclusions

Climate change will exacerbate the ecological risk of human exposure to leishmaniasis in areas north of the present range of the disease in the United States (particularly the east-central part of the country) and possibly even in parts of south-central Canada. The risk of spread is greater for the extreme A2 climate change scenario than the conservative B2 scenario. Even in the latter case, with contiguous models for dispersal of vector and reservoir species, and occupancy restricted to only the top 10% of potential habitat, the number of human individuals exposed to leishmaniasis is predicted to double by 2080. These predictions point to the importance of public health measures such as surveillance for leishmaniasis immediately north of the southern United States and, potentially, further north as disease cases are identified. Vector and reservoir control strategies should also be further investigated as part of adaptability to climate change. It is likely that other presently primarily tropical vector-borne diseases will show a similar pattern of range expansion and poleward shift due to climate change.

## Supporting Information

Figure S1 Predicted future distributions for *Lutzomyia anthophora*. (a) B2 scenario, Hadley model, 2020; (b) B2 scenario, Hadley model, 2050; (c) B2 scenario, Hadley model, 2080; (d) A2 scenario, CSIRO model, 2020; (e) A2 scenario, CSIRO model, 2050; (f) A2 scenario, CSIRO model, 2080.
(0.79 MB TIF)

Figure S2 Predicted future distributions for *Neotoma albigula*. (a) B2 scenario, Hadley model, 2020; (b) B2 scenario, Hadley model, 2050; (c) B2 scenario, Hadley model, 2080; (d) A2 scenario, CSIRO model, 2020; (e) A2 scenario, CSIRO model, 2050; (f) A2 scenario, CSIRO model, 2080.
(0.77 MB TIF)

Figure S3 Predicted future distributions for *Neotoma mexicana*. (a) B2 scenario, Hadley model, 2020; (b) B2 scenario, Hadley model, 2050; (c) B2 scenario, Hadley model, 2080; (d) A2 scenario, CSIRO model, 2020; (e) A2 scenario, CSIRO model, 2050; (f) A2 scenario, CSIRO model, 2080.
(0.67 MB TIF)

Figure S4 Predicted future distributions for *Neotoma micropus*. (a) B2 scenario, Hadley model, 2020; (b) B2 scenario, Hadley model, 2050; (c) B2 scenario, Hadley model, 2080; (d) A2 scenario, CSIRO model, 2020; (e) A2 scenario, CSIRO model, 2050; (f) A2 scenario, CSIRO model, 2080.
(0.78 MB TIF)
