# Supplementary figures and images for: Climate Change and Risk of Leishmaniasis in North America: Predictions from Ecological Niche Models of Vector and Reservoir Species
Source: PLoS Negl Trop Dis. 2010 Jan 19;4(1):e585. doi: 10.1371/journal.pntd.0000585 (PMC2799657; doi:10.1371/journal.pntd.0000585)

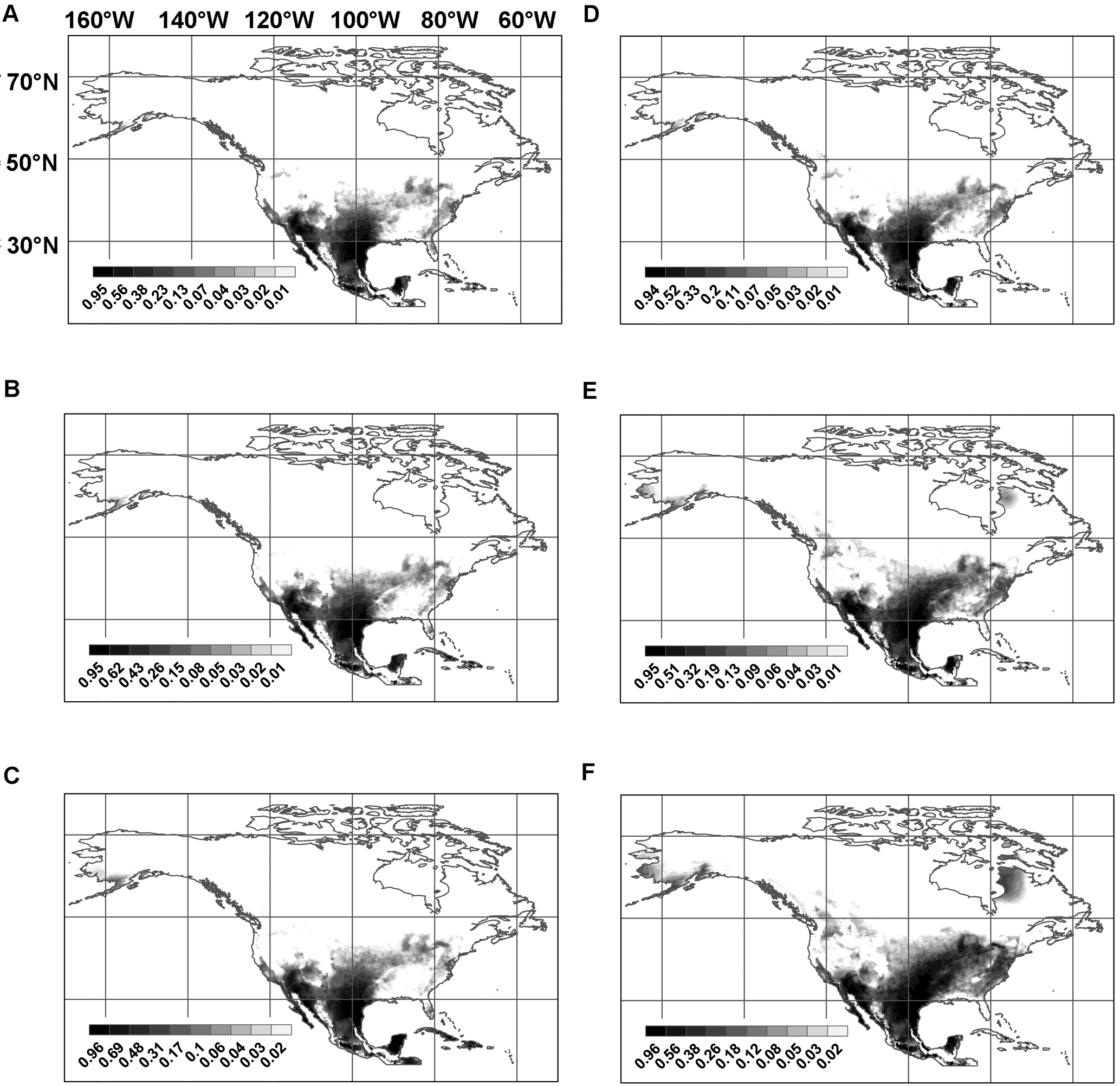

Supplement: Figure S1 — Predicted future distributions for Lutzomyia anthophora. (a) B2 scenario, Hadley model, 2020; (b) B2 scenario, Hadley model, 2050; (c) B2 scenario, Hadley model, 2080; (d) A2 scenario, CSIRO model, 2020; (e) A2 scenario, CSIRO model, 2050; (f) A2 scenario, CSIRO model, 2080. (0.79 MB TIF) [file pntd.0000585.s001.tif]

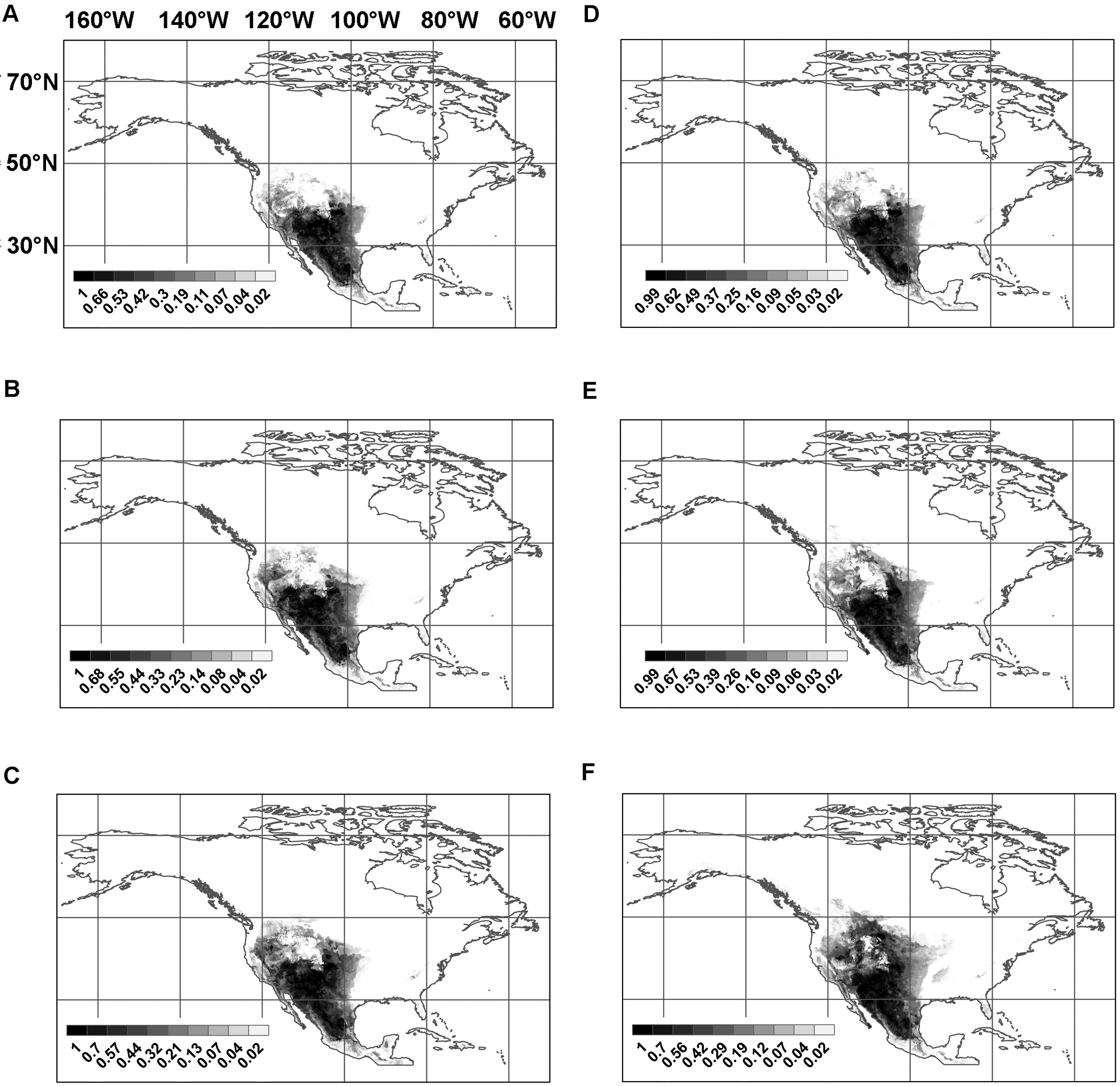

Supplement: Figure S2 — Predicted future distributions for Neotoma albigula. (a) B2 scenario, Hadley model, 2020; (b) B2 scenario, Hadley model, 2050; (c) B2 scenario, Hadley model, 2080; (d) A2 scenario, CSIRO model, 2020; (e) A2 scenario, CSIRO model, 2050; (f) A2 scenario, CSIRO model, 2080. (0.77 MB TIF) [file pntd.0000585.s002.tif]

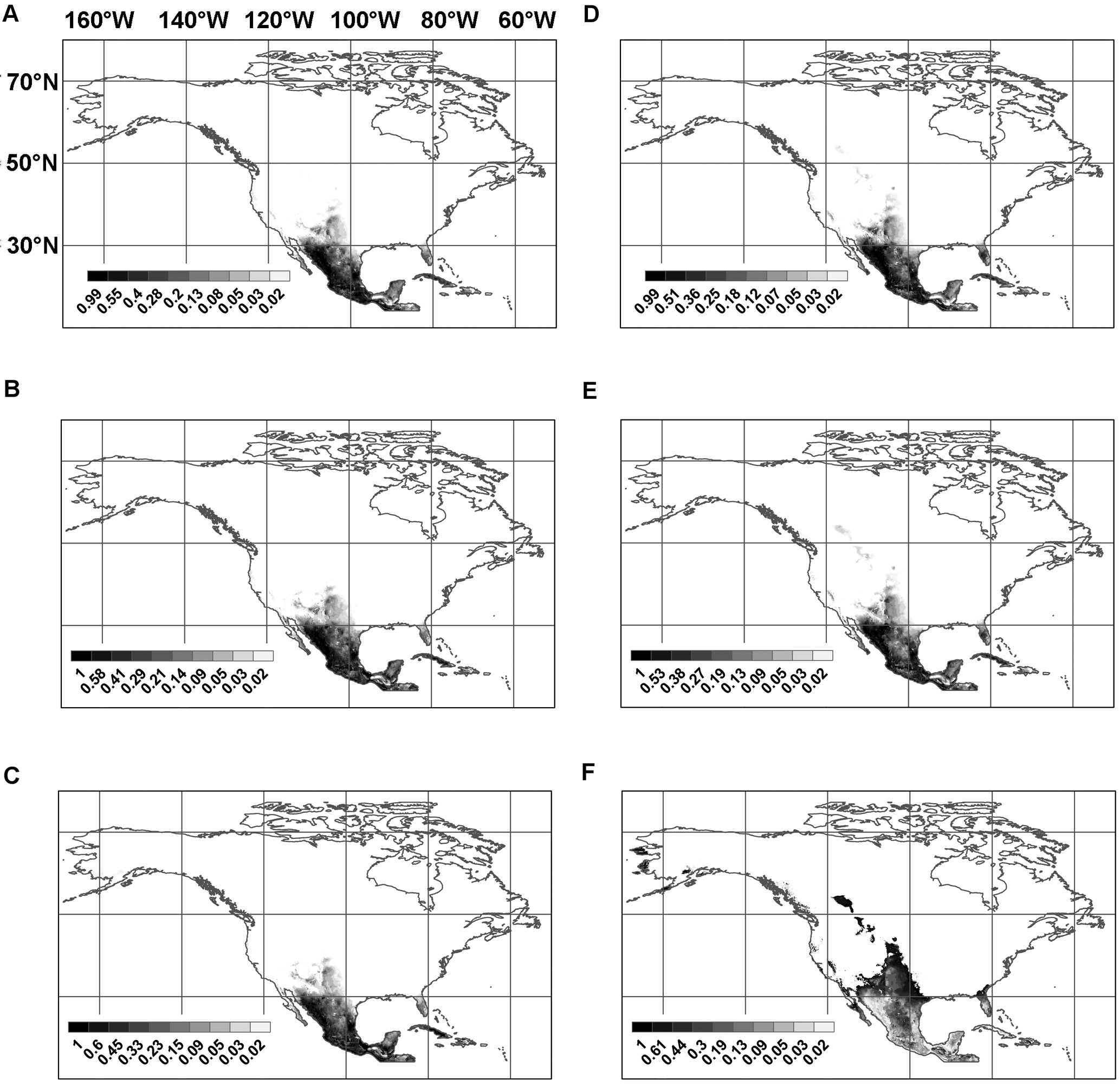

Supplement: Figure S3 — Predicted future distributions for Neotoma mexicana. (a) B2 scenario, Hadley model, 2020; (b) B2 scenario, Hadley model, 2050; (c) B2 scenario, Hadley model, 2080; (d) A2 scenario, CSIRO model, 2020; (e) A2 scenario, CSIRO model, 2050; (f) A2 scenario, CSIRO model, 2080. (0.67 MB TIF) [file pntd.0000585.s003.tif]

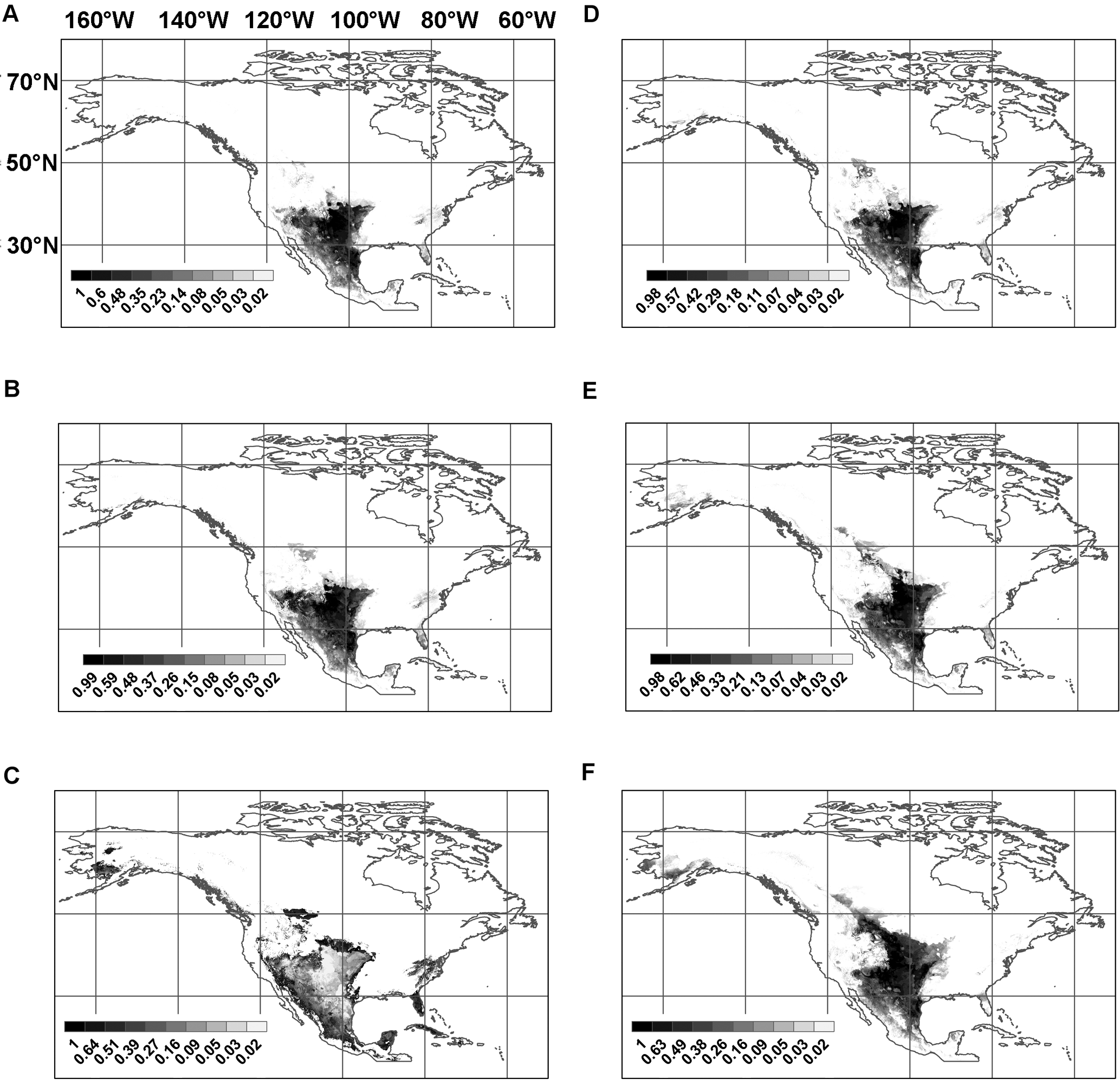

Supplement: Figure S4 — Predicted future distributions for Neotoma micropus. (a) B2 scenario, Hadley model, 2020; (b) B2 scenario, Hadley model, 2050; (c) B2 scenario, Hadley model, 2080; (d) A2 scenario, CSIRO model, 2020; (e) A2 scenario, CSIRO model, 2050; (f) A2 scenario, CSIRO model, 2080. (0.78 MB TIF) [file pntd.0000585.s004.tif]
